# The effect of an early ambulation program based on cognitive behavioral therapy for elderly patients with kinesiophobia after total hip arthroplasty

**DOI:** 10.3389/fmed.2026.1741605

**Published:** 2026-03-04

**Authors:** Yu Xie, Shuying Liu, Chunmei Li, Yan Luo, Ran Chen, Wanfei Wu, Lianyang Zhang, Qingshan Guo, Yufeng Zhao, Siru Zhou, Jun Fei, Yu Luo

**Affiliations:** 1 School of Nursing, Army Medical University (Third Military Medical University), Chongqing, China; 2 War Trauma Medical Center, State Key Laboratory of Trauma and Chemical Poisoning, Army Medical Center, Daping Hospital, Army Medical University, Chongqing, China

**Keywords:** cognitive behavioral therapy, early ambulation, fear avoidance, geriatric kinesiophobia, hip arthroplasty

## Abstract

**Objective:**

This study aimed to evaluate the effectiveness of an early ambulation program based on cognitive behavioral therapy for elderly patients with kinesiophobia after Total hip arthroplasty (THA).

**Methods:**

A quasi-experimental study design was adopted. Elderly patients with post-THA kinesiophobia admitted between December 2023 and December 2024 were enrolled and divided into a control group and an intervention group. The control group received routine health education and rehabilitation training. Whereas the intervention group received a cognitive-behavioral therapy-based early mobilization program. Postoperative data were collected, including the time of first ambulation kinesiophobia scores, pain scores, Harris scores, ADL scores. Postoperative follow-up of the patient’s kinesiophobia, pain, Harris, ADL scores was conducted at one and 3 months. Statistical analysis was conducted using *t*-test, analysis of variance and chi-square.

**Results:**

Baseline characteristics were comparable between the two groups. The intervention group demonstrated a significantly shorter time to first ambulation compared to the control group. Kinesiophobia scores improved significantly over the three postoperative time points assessed. Pain scores, Harris scores, and ADL scores also showed significant improvements. Additionally, the intervention group had a significantly shorter hospital stay and lower hospitalization costs.

**Conclusion:**

The CBT-based early mobilization intervention effectively improved health outcomes in elderly patients with post-THA kinesiophobia, offering valuable insights for clinical practice.

**Clinical trial registration:**

http://www.chictr.org.cn/showproj.aspx?proj=2400094636&lang=en, Identifier ChiCTR2400094636.

## 1 Introduction

Total hip arthroplasty (THA), which aims to completely replace the damaged hip joint with an artificial prosthesis, is a key surgical procedure for treating advanced osteoarthritis, hip fractures, and restoring joint function (1–5). Although surgical techniques continue to advance, THA is still associated with risks such as prosthesis loosening, infection, dislocation, and venous thromboembolism (6–9). Therefore, early postoperative ambulation is crucial for preventing complications and has been recommended by multiple clinical guidelines (10–12).

Early ambulation (EA) refers to the initiation of out-of-bed activities as soon as possible within 24 h after surgery (13). However, clinical practice lags behind guideline recommendations, with patients generally experiencing delays in their first ambulation attempt (14). In addition to physiological factors, kinesiophobia has been identified as an independent psychological predictor hindering early ambulation. Kinesiophobia is defined as an excessive, irrational fear of movement due to anticipated pain or reinjury (15, 16). Approximately 50% of THA patients are affected by kinesiophobia (17), and its severity increases with age (18, 19), significantly impacting postoperative pain and functional recovery.

Cognitive behavioral therapy (CBT) is an effective approach for breaking the fear-avoidance cycle. By modifying catastrophic thinking and maladaptive behaviors related to pain and movement, CBT has demonstrated efficacy in alleviating kinesiophobia in contexts such as chronic pain and rehabilitation after total knee arthroplasty (20, 21). However, existing studies often treat CBT as a standalone psychological intervention. There is a lack of a structured, operable, and integrated rehabilitation protocol that deeply incorporates core CBT techniques into the postoperative early ambulation pathway and is specifically designed for elderly THA patients. This lack of integrated protocols limits the systematic application of CBT in routine THA clinical rehabilitation.

To address the lack of integrated protocols, the present study drew on the Fear-Avoidance Model (22) to define the core problem of kinesiophobia and incorporated insights from Protection Motivation Theory (23), specifically regarding threat appraisal and coping appraisal, to design an early ambulation protocol that integrates safety-focused activity guidance with cognitive-behavioral strategies. The CBT based early ambulation protocol not only provides standardized activity instructions but also emphasizes alleviating kinesiophobia through psychological intervention, thereby fundamentally breaking the fear-avoidance cycle and ultimately promoting early patient mobilization. To evaluate the practical effectiveness of the CBT based early ambulation protocol, the present study aimed to conduct a preliminary clinical trial to assess its effects on improving early ambulation behavior, reducing kinesiophobia, and enhancing functional recovery in elderly THA patients.

## 2 Methods

### 2.1 Participants

This study included elderly patients with kinesiophobia following total hip arthroplasty (THA), who were admitted to the Department of Trauma Surgery and the Department of Joint Surgery at a tertiary general hospital in Chongqing, China, from December 2023 to December 2024. Patients who met the inclusion and exclusion criteria were enrolled consecutively: those admitted between December 2023 and June 2024 were allocated to the control group, and those admitted between July 2024 and December 2024 were allocated to the intervention group.

Inclusion criteria were: (1) elderly patients undergoing primary unilateral THA with stable postoperative vital signs; (2) a score > 37 on the Chinese version of the Tampa Scale for Kinesiophobia (TSK) within 24 h after surgery (24); (3) age ≥ 65 years; (4) conscious, able to communicate effectively, and free from mental illness; (5) voluntary participation with written informed consent. Exclusion criteria were: (1) cognitive impairment or consciousness disorders; (2) severe restriction of lower limb movement unrelated to THA; (3) inability to communicate normally; (4) withdrawal during the study period or non-cooperation. During the study period, 120 patients scheduled for primary THA were assessed. Of these, 56 were excluded (54 did not meet the inclusion criteria; 2 declined to participate). Consequently, 64 eligible patients were enrolled and allocated sequentially to the control group (*n* = 31) and intervention group (*n* = 33).

**Figure 1:**
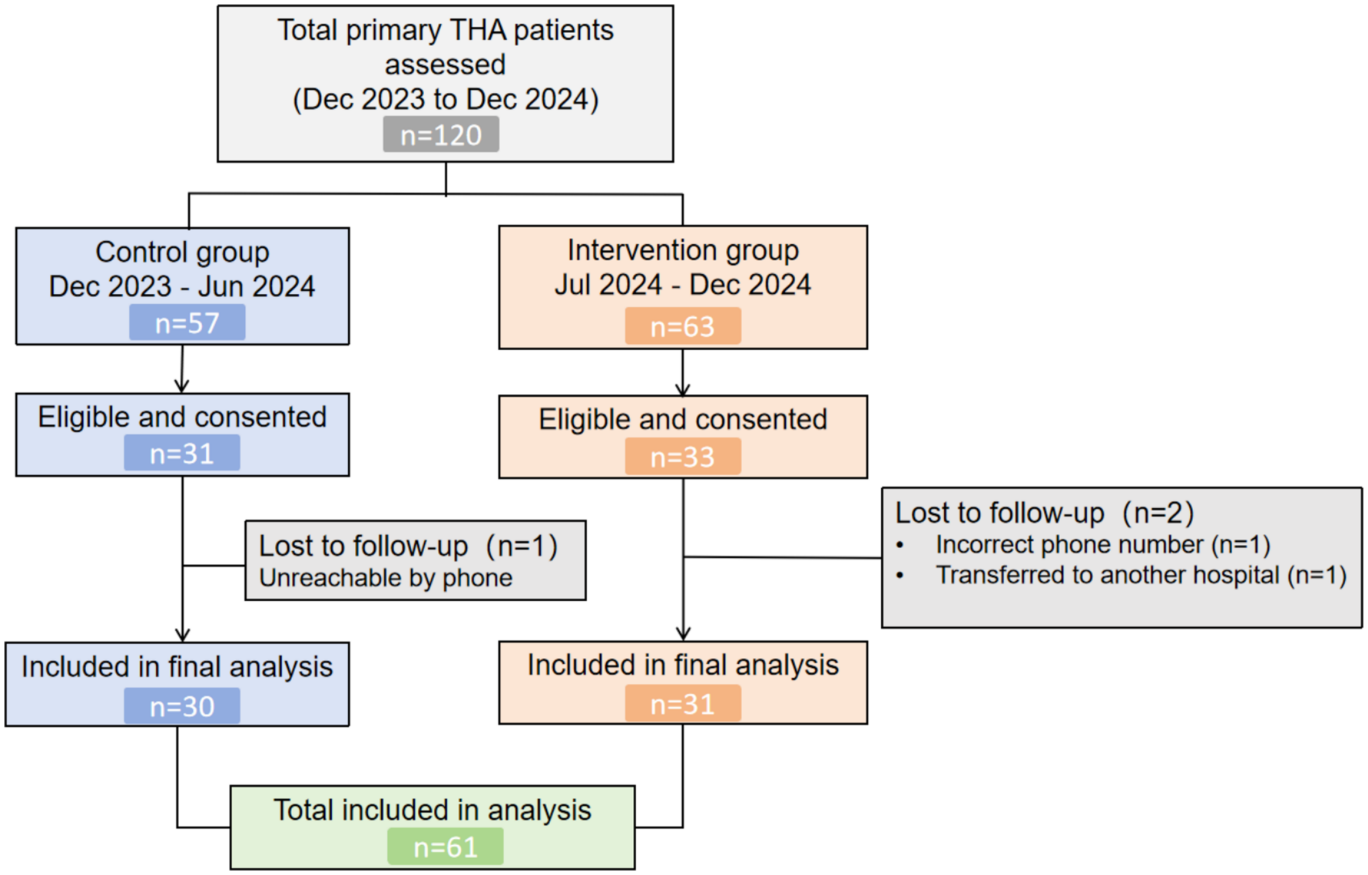
Participant flow diagram.

During follow-up, 3 patients were lost (control group: *n* = 1 due to incorrect contact information; intervention group: *n* = 2, including one with incorrect contact information and one transferred to a rehabilitation hospital). The final analysis included 61 patients who completed the study (Figure 1).

The study protocol was reviewed and approved by the Ethics Committee of the Army Medical Center of the Chinese People’s Liberation Army (Approval No. [2020] 194–01) and was prospectively registered with the Chinese Clinical Trial Registry (Identifier: ChiCTR2400094636). All participants provided written informed consent prior to enrollment. Baseline characteristics of the participants are presented in Table 1.

**Table 1:** Baseline comparison results of general information of research subjects.

Indicator/grouping	Intervention group (*n* = 31)	Control group (*n* = 30)	*t*-value (Cohen’s d)	*χ^2^*-value	*p* value
Age (years)	82.10 ± 9.61	81.77 + 7.96	0.15 (0.94)		0.885*
BMI (kg/m^2^)	21.74 ± 3.73	21.11 ± 3.50	0.67 (0.17)		0.504*
Gender (%)				0.84	0.360^#^
Male	8 (25.8)	11 (36.7)			
Female	23 (74.2)	19 (63.3)			
Marital status (%)				0.138	0.710^#^
Married	18 (58.1)	16 (53.3)			
Unmarried	0	0			
Divorced or widowed	13 (41.9)	14 (46.7)			
Educational level (%)				0.92	0.821^#^
Elementary school and below	19 (61.3)	21 (70.0)			
Junior high school	5 (16.1)	3 (10.0)			
High school or vocational school	5 (16.1)	5 (16. 7)			
University and above	2 (6.5)	1 (3.3)			
Smoking history (%)				0.00	1.000^#^
Yes	3 (9.7)	3 (10.0)			
No	28 (90.3)	27 (90.0)			
Drinking history (%)				0.001	0.977^#^
Yes	1 (3.2)	2 (6.7)			
No	30 (96.8)	28 (93.3)			
Preoperative fear score	45.45 ± 3.86	43.63 ± 4.96	1.60 (0.41)		0.115*
Preoperative pain score	2.65 ± 1.11	2.53 ± 1.01	0.41 (0.11)		0.682*
Preoperative Harris	19.77 ± 5.16	19.80 ± 5.81	0.02 (0.01)		0.985*
Preoperative ADL score	34.19 ± 7.65	34.00 ± 8.35	0.09 (0.02)		0.925*
Preoperative complications	1.77 ± 0.43	1.63 ± 0.49	1.20 (0.31)		0.235*

Data are presented as *n* (%) or M ± SD. M, mean; SD, standard deviation. *Indicates that the statistic is a *t*-value, ^#^representing statistical measures as chi square value.

### 2.2 Tests administered

Data were collected at four time points: admission, discharge, one month post-surgery, and 3 months post-surgery. The following outcomes were assessed: (1) time to first ambulation (days); (2) kinesiophobia, assessed using the TSK score (24) (the TSK is a 17-item scale with total scores ranging from 17 to 68, with a score >37 indicating the presence of kinesiophobia and higher scores reflecting more severe fear of movement); (3) hip joint function, assessed using the Harris Hip Score (25); (4) pain intensity, measured using the Numeric Rating Scale (NRS) (26) (ranging from 0 for no pain to 10 for worst possible pain); (5) activities of daily living, assessed using the Barthel Index (27) (which evaluates independence in 10 ADLs with total scores ranging from 0 to 100, where scores ≤20 indicate complete dependence); and (6) length of hospital stay (days) and hospitalization expenses (CNY).

### 2.3 Procedure

#### 2.3.1 Study design

This study employed a quasi-experimental design with sequential time-based grouping (control group followed by intervention group). Although randomized controlled trials are considered the gold standard for causal inference, strict randomization of systematic clinical interventions, such as cognitive behavioral therapy following total hip arthroplasty, often faces ethical and practical feasibility constraints. This design enabled the natural implementation and evaluation of the intervention in a real-world clinical setting, an approach widely adopted in health services research.

It should be noted that due to the sequential nature of the grouping, outcome assessors could not be fully blinded. However, all outcome measures, including TSK scores, Harris Hip Scores, and time to first ambulation, were collected uniformly by an independent assessor (SL) who was not involved in clinical intervention delivery. The intervention was delivered by CL and YL, who underwent unified training and followed a structured operational manual to ensure standardization of both intervention content and procedures.

#### 2.3.2 Interventions

##### 2.3.2.1 Control group

During the patient’s hospitalization, nursing measures for patients undergoing THA included perioperative health education, early postoperative functional exercise, dietary guidance, information on the effects and usage of common medications, and prevention of complications. The control group received routine care without structured intervention at specific time points.

##### 2.3.2.2 Intervention group

The theoretical framework and corresponding intervention measures are presented in Table 2. The intervention program was divided into six phases: pre-implementation preparation, admission day, 24 h pre-surgery, surgery day, postoperative day 1, and discharge day. Specific measures included cognitive and behavioral assessments, problem identification, and individualized rehabilitation interventions based on patient evaluations. The detailed phase-specific intervention measures are provided in Table 3. The intervention group received the CBT-based early ambulation program according to the scheduled phases outlined in Table 3.

**Table 2:** Theoretical core components, themes, and corresponding intervention measures.

Core components	Themes	Corresponding intervention measures
Threat assessment	Severity	① NRS Pain Assessment; assessment of vital signs and physical function, combined with postoperative imaging results; ② Cognitive Assessment: identification of the types and causes of patients’ concerns about secondary injury; ③ Cognitive and Behavioral Intervention: health education to change the perception that early ambulation increases the risk of secondary injury; nurses providing one-on-one health education on the importance and safety of early ambulation and functional exercise.
	Susceptibility	① Assessment of patient’s self-care ability, muscle strength, nutrition, and fall risk; ② Cognitive Assessment: identification of the reasons why patients worry about being more susceptible to injury; ③ Cognitive and Behavioral Intervention: health education, providing nutritional support and functional exercise guidance.
	Internal external rewards	① Establishment of a multidisciplinary intervention management team for kinesiophobia following total hip arthroplasty; ② Cognitive Assessment: inquiry into patients’ current attitudes toward early ambulation, progress in current rehabilitation exercises, and level of family support; ③ Cognitive and Behavioral Intervention: nursing plan is adjusted in real-time based on patients’ periodic feedback and assessment results.
Coping assessment	Response efficacy	① One-on-one nurse education on the importance and safety of early ambulation and functional exercise; ② Sharing successful kinesiophobia recovery cases, peer support, role modeling, and inviting senior patients with good recovery outcomes to share experiences.
	Self-efficacy	① Patient Self-efficacy and Understanding Assessment Upon regaining consciousness, inquire about the patient’s understanding of early ambulation protocols post-surgery and assess their self-efficacy; ② Early Ambulation Preparation and Psychoeducation Conduct preparatory education for early ambulation, systematically address potential difficulties the patient may face, and foster the patient’s belief in their ability to successfully complete early ambulation activities; ③ Immediate Postoperative Bed Training and Environmental PreparationInitiate in-bed exercises once alert post-anesthesia. Ensure ward prepared, consistent staff instructions.
	Reaction cost	① Cognitive Assessment (Burden on Caregivers) Identify the patient’s concerns about increasing the burden on family caregivers due to their need for early ambulation; ② Cognitive and Behavioral Intervention (Stress Reduction) Provide health education aimed at alleviating patient stress related to caregiver burden; ③ Collaborative Long-term Rehabilitation Planning Co-create a long-term rehabilitation plan with the patient.

**Table 3:** Phase-specific intervention measures of the CBT-based early ambulation program.

Time phase	Assessment	Cognitive intervention	Rehabilitation measures
Pre-implementation	—	—	Multidisciplinary team; roles clarified; personalized plans
Admission day	TSK; demographics; cognitive questionnaire	*Problem ID*: Identify catastrophic thoughts/fears. *Education*: 1-on-1 (kinesiophobia definition, impact, early ambulation importance/safety); checklist for comprehension; videos (muscle relaxation, success stories). *Mobilization guidance*: Sit up (gradual head elevation, no support) → bedside sit (healthy side, feet flat) → bedside stand (assisted, even weight-bearing) → walking (sequence: stable start→walker first→surgical limb→healthy limb; upright posture, forward gaze)	*Daily exercises*: Progressive muscle relaxation (20-30 min,1x/d); pursed lip breathing (30 sets,3x/d); ankle pumps (10 min,3x/d); quadriceps/hamstring isometrics (50–100 reps,3x/d); clinical execution form
24 h Pre-surgery	Reassess cognition; previous education mastery	Share success stories; reinforce mobilization guidance; review preoperative movement familiarization	Continue daily exercises
Surgery day	Lines/drains/strength; THA understanding; rehab progress; family support	Identify/address difficulties; ensure consistent instructions	*Nutrition*: Post-op: head up, 30 mL water → swallow test (grade≥2,no nausea) → ONS @2 h → normal diet @4 h. Continue daily exercises
Post-op day 1	*Pre-ambulation*: Vital signs, stability (delay if abnormal). *Cognitive*: Ambulation step mastery	Address knowledge gaps; psychological support for fearful patients	*Preparation*: Environment (dry, clear floor, minimal traffic); equipment (walker, non-slip shoes); patient (analgesia, secure lines, remove drains). *Mobilization*: Supervised sequence: sit up→bedside sit→stand→walk; monitor: dizziness,fatigue,sweating,nausea,palpitations,chest tightness; if pain worsens→bed rest
Discharge day	Overall status	Identify problems; targeted reinforcement	—

ONS, oral nutritional supplements.

Abbreviations for frequency and dosage: x/d, times per day (e.g., 1x/d = once daily, 3x/d = three times daily); min, minutes; reps, repetitions; mL, milliliters; h, hours; @, at (e.g., @2 h = at 2 h post-operation).

Symbols: → indicates sequence of steps (e.g., sit up → bedside sit → stand → walk).

“Daily exercises” refers to the standard in-bed exercise protocol detailed on Admission Day (progressive muscle relaxation, pursed lip breathing, ankle pumps, and isometric exercises). All interventions are delivered by trained nursing staff.

### 2.4 Statistical procedures

#### 2.4.1 Sample size calculation

Sample size was estimated using G*Power software (version 3.1.9.7). Based on previous similar studies and preliminary findings from this research (28), using time to first ambulation as the primary outcome, the estimated effect size (*δ*) was approximately 3.34. With a significance level of *α* = 0.05 (two-tailed) and statistical power of 1-*β* = 0.80, using a paired *t*-test model (within-group comparison), the initial calculated sample size was approximately 5 cases for the intervention group. Accounting for an estimated attrition rate of 20%, the intervention group required at least 6 elderly participants with kinesiophobia.

It should be noted that the above calculation, based on a within-group framework, was intended to provide a theoretical minimum sample size reference for the intervention group, rather than a rigid target for between-group comparisons. As this was a feasibility study, the final sample size was primarily determined by pragmatic considerations: to maximize real-world representativeness and ensure external validity, we consecutively enrolled all eligible patients presenting at our center throughout the study period (December 2023 to December 2024) (29). Ultimately, 30 patients were included in the control group and 31 in the intervention group, yielding a total sample of 61 participants. This sample size is consistent with recommendations for feasibility studies and is comparable to those reported in similar studies in this field (30–32). Importantly, statistically significant differences were observed in the primary outcome (time to first ambulation) and multiple secondary outcomes, confirming that the current sample size was sufficient to detect clinically meaningful effects. According to recent methodological literature, post-hoc power analysis is not recommended and was therefore not performed.

#### 2.4.2 Statistical analysis

Statistical analysis was conducted using SPSS version 27.0 software. Count data were presented as frequencies and percentages, while continuous data were expressed as mean ± standard deviation or median, as appropriate. For comparisons between two groups, independent samples *t*-tests were used for normally distributed data, and Mann–Whitney U tests were used for non-normally distributed data. Categorical variables were analyzed using the chi-square test. Repeated measures analysis of variance (ANOVA) was conducted to compare outcomes between the two groups at four time points: baseline (admission), discharge, one month post-surgery, and 3 months post-surgery. To account for multiple comparisons across different time points and outcome measures, *p*-values were adjusted using the Bonferroni correction.

## 3 Results

### 3.1 Comparison of baseline characteristics between the two groups

No statistically significant differences were observed between the two groups in baseline characteristics, including age, gender, body mass index (BMI), admission NRS score, education level, marital status, comorbidities, smoking status, and alcohol consumption (all *p* > 0.05) (Table 1).

### 3.2 Comparison of first mobilization time, hospitalization duration, and hospitalization costs

As shown in Table 4, the time to first ambulation was significantly shorter in the intervention group (1.23 ± 0.72 days) compared to the control group (3.30 ± 2.15 days, *p* < 0.001). The intervention group also had significantly shorter hospital stays (7.35 ± 1.78 days vs. 9.83 ± 3.36 days, p < 0.001) and lower hospitalization expenses (3704.91 ± 605.24 CNY vs. 4761.83 ± 1201.51 CNY, p < 0.001).

**Table 4:** Comparison of first mobilization time, length of hospitalization, and hospitalization expenses after THA between the two groups.

Indicator/Grouping	Intervention group (*n* = 31)	Control group (*n* = 30)	Cohen’s d	95% CI	*t-*value	*p* value
First time of getting out of bed activity (d)	1.23 ± 0.72	3.30 ± 2.15	1.30	[0.75,1.85]	5.08	<0.001
Length of hospitalization (d)	7.35 ± 1.78	9.83 ± 3.36	0.93	[0.39,1.45]	3.61	<0.001
Hospitalization expenses (CNY)	3704.91 ± 605.24	4761.83 ± 1201.51	1.12	[0.57,1.65]	4.36	<0.001

Data are presented as M ± SD. d, days; CNY, Chinese Yuan.

### 3.3 Comparison of outcome measures between the two groups at different time points

**Figure 2:**
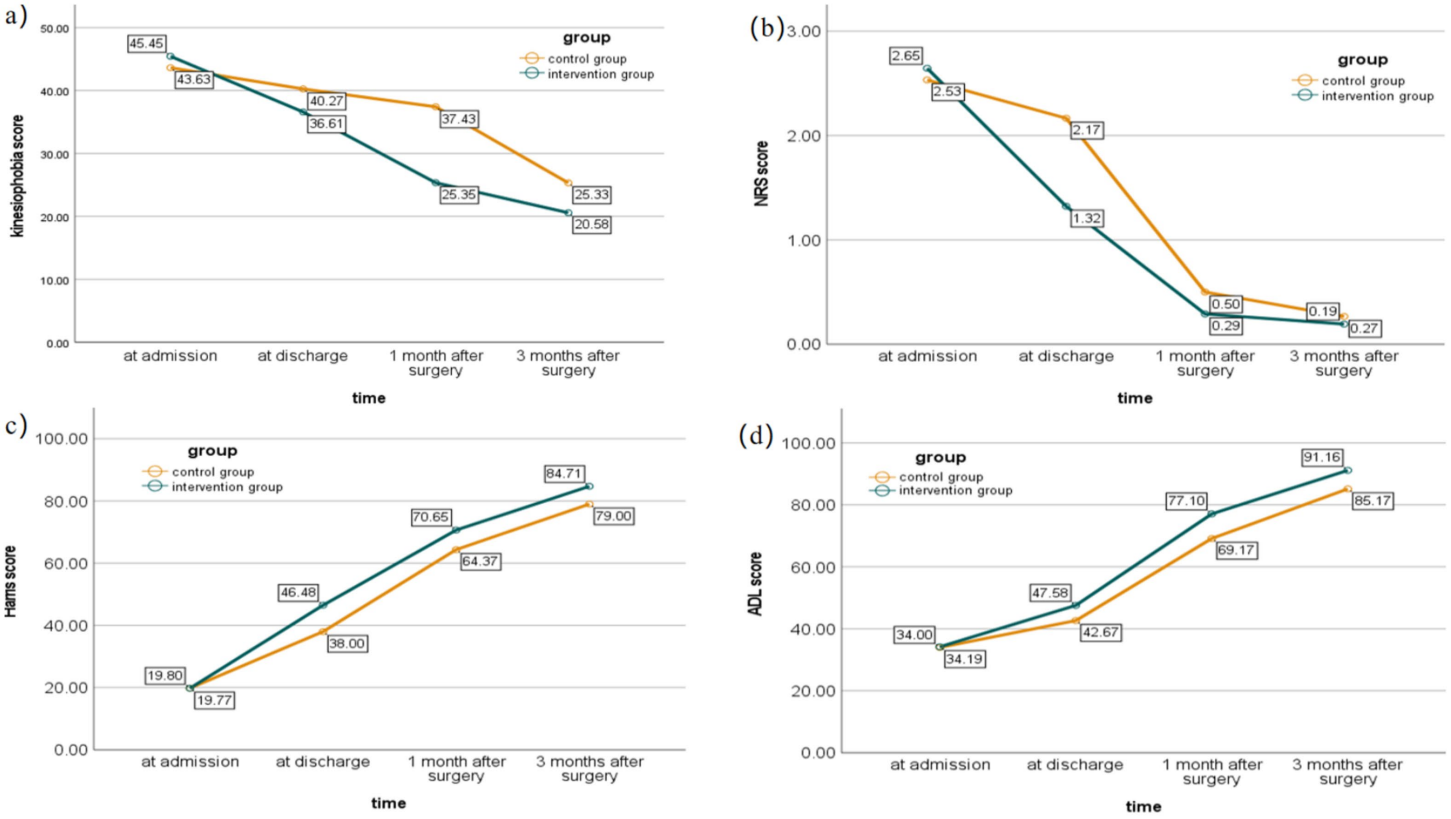
Comparison of scores between the intervention group and control group at different time points. **(a)** Comparison of kinesiophobia scores between the intervention group and control group at different time points. **(b)** Comparison of NRS scores between the intervention group and control group at different time points. **(c)** Comparison of Harris scores between the intervention group and control group at different time points. **(d)** Comparison of ADL scores between the intervention group and control group at different time points.

At baseline, no statistically significant differences were observed between the two groups for any of the outcome measures (*p* > 0.05) (Table 3). Repeated measures ANOVA revealed significant main effects of time for all outcome measures (*p* < 0.05) (Table 5). Significant time × group interaction effects were observed, indicating that the intervention group showed greater improvements over time compared to the control group. Specifically, at discharge, 1 month post-surgery, and 3 months post-surgery, the intervention group demonstrated more pronounced reductions in kinesiophobia and pain scores, as well as greater improvements in hip function and activities of daily living (all *p* < 0.05) (Figure 2). These preliminary findings provide initial evidence that the CBT-based early ambulation program may be associated with reduced kinesiophobia and improved functional recovery in elderly THA patients, although these results require confirmation in larger studies.

**Table 5:** Comparison of kinesiophobia scores, NRS scores, Harris scores, and ADL scores between two groups of patients at different time points within each group.

Grouping/Time point	On admission	At discharge	1 month after surgery	3 months after surgery	Time	Group	Time *Group
					*p* value (*F*, η^2^)	*p* value (*F*, η^2^)	*p* value (*F*, η^2^)
Kinesiophobia scores							
Intervention group	45.45 ± 3.86	36.61 ± 4.13	25.35 ± 7.59	20.58 ± 5.66	<0.001 (*F* = 224.45, η^2^ = 0.79)	<0.001 (*F* = 17.03, η^2^ = 0.22)	<0.001 (*F* = 19.13, η^2^ = 0.25)
Control group	43.63 ± 4.96	40.27 ± 3.81	37.43 ± 8.35	25.33 ± 8.21			
NRS scores							
Intervention group	2.65 ± 1.11	1.32 ± 0.87	0.29 ± 0.64	0.19 ± 0.40	<0.001 (*F* = 171.71, η^2^ = 0.74)	0.047 (*F* = 4.12, η^2^ = 0.07)	<0.001 (*F* = 5.82, η^2^ = 0.09)
Control group	2.53 ± 1.01	2.17 ± 0.38	0.50 ± 0.82	0.27 ± 0.45			
Harris scores							
Intervention group	19.77 ± 5.16	46.48 ± 5.37	70.65 ± 6.66	84.71 ± 8.61	<0.001 (*F* = 1312.45, η^2^ = 0.96)	<0.001 (*F* = 20.11, η^2^ = 0.25)	<0.001 (*F* = 5.73, η^2^ = 0.09)
Control group	19.80 ± 5.81	38.00 ± 6.92	64.37 ± 8.00	79.00 ± 7.04			
ADL scores							
Intervention group	34.19 ± 7.65	47.58 ± 4.63	77.10 ± 9.29	91.16 ± 7.40	<0.001 (*F* = 670.07, η^2^ = 0.92)	<0.001 (*F* = 11.57, η^2^ = 0.16)	0.035 (*F* = 2.93, η^2^ = 0.05)
Control group	34.00 ± 8.35	42.67 ± 3.88	69.17 ± 12.04	85.17 ± 11.33			

All data (including kinesiophobia scores, NRS scores, Harris scores, ADL scores) are presented as M ± SD.

## 4 Discussion

This is an exploratory, effectiveness-oriented study grounded in the Fear-Avoidance Model and Protection Motivation Theory. It aims to preliminarily evaluate the feasibility and potential efficacy of a cognitive behavioral therapy-based early ambulation program for elderly patients undergoing total hip arthroplasty. Observed data suggest that this program holds promise for reducing kinesiophobia and promoting functional recovery, warranting further investigation.

### 4.1 Interpretation of findings in relation to theoretical models

In this study, the intervention group demonstrated more favorable trends than the control group in time to first ambulation, length of hospital stay, and pain and kinesiophobia scores at discharge. These observations suggest that integrating CBT components into postoperative rehabilitation may help influence the fear-avoidance psychological cycle. Through health education, cognitive restructuring, and progressive activity scheduling, the program was associated with a trend toward reduced patient concerns regarding the perceived severity and susceptibility of adverse events related to early ambulation. As fear levels decreased, patients’ willingness to mobilize showed an improving trend. Enhanced feelings of being cared for, as reported by patients and their families, may be associated with earlier initiation of functional exercise. This interpretation is consistent with findings from Kamp et al. (33), who reported that increased external support benefits hip and knee functional recovery. However, it is important to clarify that this study is a pragmatic preliminary effectiveness trial, not a mechanistic study. Therefore, any interpretations related to theoretical mechanisms remain hypothetical, as no mediating variables were measured.

Notably, nutritional support was a standard baseline measure common to both groups. The advantage of the intervention group lay in the integration of nutritional support with rehabilitation goals at both cognitive and behavioral levels through the CBT framework, thereby enhancing implementation systematization and adherence. The nutritional support provided post-admission contributed to strengthening patients’ lower limb muscle strength, laying the foundation for early ambulation. This study provides preliminary empirical support for the positive effects of a CBT-based early ambulation program on patient health outcomes.

### 4.2 Connections and contributions to existing research

Previous studies have established that kinesiophobia is a significant barrier to long-term recovery in patients following total hip arthroplasty. The retrospective study by Al-Amiry et al. (34) confirmed that 6–8 years post-THA, patients with kinesiophobia exhibited lower hip function scores and higher rates of walking aid dependence compared to those without kinesiophobia. However, that study did not explore intervention strategies targeting kinesiophobia itself. Subsequent research has provided directions for intervention. A systematic review by Rhamelani et al. (35) indicated that early ambulation after orthopedic surgery constitutes a comprehensive intervention; the five categories of interventions identified (range of motion interventions, progressive muscle relaxation, weight-bearing interventions, positioning interventions, and neuromuscular electrical stimulation) all contributed to shortened hospital length of stay. Among these, interventions based on progressive muscle relaxation demonstrated potential for alleviating short-term kinesiophobia, suggesting that integrating psychological components into traditional rehabilitation may enhance therapeutic effects.

Regarding active interventions for kinesiophobia, relatively mature CBT frameworks have been established in other orthopedic surgical populations. For instance, Scarone et al. (36) randomized 150 patients following lumbar fusion surgery and found that those receiving CBT (including pain education, cognitive restructuring, etc.) combined with exercise showed significantly greater improvements in disability reduction, pain relief, and quality of life compared to the control group receiving only traditional health education and exercise, with effects sustained for at least 12 months. Studies by Abbott et al. (37) (*n* = 107) and Cai et al. (28) (*n* = 108) further confirmed the effectiveness of psychomotor therapy and cognitive-behavioral interventions in improving function, pain, and kinesiophobia levels in patients following lumbar fusion surgery and total knee arthroplasty, respectively.

It should be noted that the above evidence originates from other orthopedic surgical populations and does not constitute direct evidence for THA patients. This validated CBT intervention model has not yet been systematically applied to elderly THA patients with kinesiophobia. Several studies provide indirect support for this approach: Emporiti et al. (38) implemented preoperative action observation and motor imagery training in 80 THA patients and demonstrated that preoperative psychological preparation benefits postoperative activity, which aligns with the preoperative cognitive education concept of the present study. Research by Vasconcelos et al. (39, 40) and Smith et al. (41) supports the positive role of psychological interventions in hip functional recovery from the perspectives of physiological mechanisms (the association between muscle strength and kinesiophobia) and clinical efficacy, respectively. Accordingly, the present study provides a foundational reference for future research in this field.

### 4.3 Study limitations and future directions

This study has several limitations. First, the quasi-experimental design with sequential group allocation is susceptible to temporal bias and precludes assessor blinding. Although we controlled for key covariates and used independent assessors for outcome measurement, unmeasured systematic changes during the intervention period may have confounded effect estimates. Second, the single-center design within a specific cultural context limits the generalizability of our findings, as local factors such as family caregiving patterns and healthcare delivery models may have influenced outcomes. Third, the relatively short follow-up period of 3 months precludes assessment of longer-term effect sustainability. Fourth, selection bias may be present, with a 46.7% exclusion rate potentially limiting generalizability. Fifth, surgical complexity metrics such as operative time, blood loss, and surgical approach were not recorded or adjusted for, representing a potential source of unmeasured confounding. Additionally, the modest sample size, reliance on self-report measures, and elevated risk of Type I error due to multiple comparisons mean that positive findings should be viewed as exploratory.

To address the limitations outlined above, future research should employ factorial or component-dismantling designs to isolate the effects of specific intervention elements, routinely collect and adjust for potential confounders including surgical complexity, conduct multi-center randomized controlled trials with objective measures such as activity trackers and functional performance tests, plan formal mediation analyses to explore mechanisms of action, and perform *a priori* sample size calculations based on the effect sizes observed in the present study. The present study provides preliminary groundwork and methodological reference to inform such future research.

## 5 Conclusion

This study provides preliminary evidence that the integrated CBT-based early ambulation program may support early mobilization and reduce kinesiophobia in elderly patients following THA. These findings offer a foundational reference for future research and for the development of targeted rehabilitation strategies in this population.

## Data Availability

The raw data supporting the conclusions of this article will be made available by the authors, without undue reservation.
